# Ready-to-eat food intake associates with PHQ-9-based depression in US adults: a cross-sectional study

**DOI:** 10.1186/s12889-025-22930-x

**Published:** 2025-05-13

**Authors:** Maximilian Andreas Storz

**Affiliations:** Department of Internal Medicine II, Centre for Complementary Medicine, Medical Center - University of Freiburg, Faculty of Medicine, Freiburg, Germany

**Keywords:** Ready-to-eat foods, PHQ-9, Depression, NHANES, Public health, Nutritional neurosciences, Nutrients

## Abstract

**Background:**

Depressive disorders are a contemporary global public health problem and amongst the leading causes of disability worldwide. Ready-to-eat foods require minimal preparation time and are designed to maximize consumer convenience while minimizing consumer efforts. Nutritional properties of ready-to-eat foods, such as their high saturated fat and sodium content, have been linked to depression. Studies from the United States (US) of America investigating the association between ready-to-eat food intake frequency and depression are scarce, although North America is currently the largest ready-to-eat food market.

**Methods:**

Using crude and multivariate logistic regression models, this study explored potential associations between self-reported ready-to-eat food intake frequency and PHQ-9-based depression in US adults aged 20 years or older based on data from the cross-sectional National Health and Nutrition Examination Survey (2011–2018). Depression was assessed with the PHQ-9 questionnaire, a validated questionnaire and measure of reference in epidemiological depression research.

**Results:**

Data from *n* = 8,689 participants was analyzed. Participants with PHQ-9-based depression tended to be female, never married or separated and were characterized by a lower annual household income. Crude and adjusted multivariate logistic regression analyses revealed a significant association between ready-to-eat food intake frequency and depression. For each additional ready-to-eat food, the odds for PHQ-9-based depression increased by a factor of 1.014 (CI: 1.002–1.026; *p* = 0.022) after adjustment for sociodemographic and lifestyle characteristics. Compared to those who did not report any ready-to-eat foods, participants with an average intake frequency of ≥ 1 per day had a significantly higher likelihood of depression (OR: 2.02, CI: 1.18–3.43; *p* = 0.011) after adjustment for sociodemographic and lifestyle characteristics.

**Conclusions:**

Ready-to-eat food intake frequency is associated with PHQ-9-based depression in the NHANES. Given the high popularity of ready-to-eat foods in the US, these findings have important public health nutrition implications, and warrant additional research and targeted interventions to promote healthier meals sold by food outlets.

**Supplementary Information:**

The online version contains supplementary material available at 10.1186/s12889-025-22930-x.

## Background

Ready-to-eat foods have become increasingly popular in recent decades and are now an integral part of many people’s eating habits [1–3]. These foods are designed to require minimal preparation time, thereby minimizing the effort for the consumer while maximizing convenience [4, 5]. Ready-to-eat foods are therefore of particular interest to working and younger people [5].

The European Commission defines ready-to-eat foods as foods “intended by the producer or manufacturer for direct human consumption without further heating or other processing necessary to destroy the relevant micro-organisms or to reduce them to an acceptable level” [6]. Other definitions also explicitly add ready-made frozen foods to this food category, and state that these foods can be prepared and served quickly as they require no further preparation prior to consumption, except for thawing or heating [7, 8]. The most commonly consumed ready-to-eat foods include pre-packaged sandwiches, pre-washed salads with dressing, cooked meats or smoked fish, desserts and sweet dishes, as well as various cheese preparations and soups [2, 5].

Ready-to-eat foods are now ubiquitously available to the general public, e.g., in supermarkets, fast-food restaurants, gas stations and at schools, universities and other educational facilities [5, 9], which may have important implications for public health. Epidemiological studies suggested that the regular consumption of ready-to-eat foods is associated with an increased risk of obesity and type-2-diabetes [4, 10]. Both are, in turn, established risk factors for depressive disorders [11, 12]. As such, ready-to-eat food intake could be indirectly linked to depression.

Depressive disorders are a contemporary public health problem and amongst the leading causes of disability worldwide [13, 14]. The global prevalence of depression is increasing, which has a major impact on economies and people’s quality of life [14, 15].

Associations between diet quality and mental illness have been subject to intensive research [16, 17]. The regular consumption of unprocessed plant-based foods may protect from depression, whereas a frequent consumption of highly processed, animal-based, high-fat foods may increase the likelihood of depressive disorders [16, 17]. Ready-to-eat foods are generally characterized by a high (saturated) fat and sodium content [18, 19], with the former potentially contributing to neuroinflammation and metabolic dysregulation [20, 21]. Potential associations between the consumption of ready-to-eat foods and depression thus deserve additional consideration [22]. Liu et al. investigated ready-to-eat food intake in 2579 Chinese college students, and emphasized that a lower intake markedly decreased the likelihood of depression (Odds Ratio (OR): 0.70 (Confidence Interval (CI): 0.57–0.86), *p* < 0.001) [22]. Comparable investigations in North American populations are still scarce, although the US is currently the largest ready-to-eat food market [23].

We sought to explore potential associations between ready-to-eat food intake frequency and depression in US adults analyzing nationally representative data from the National Health and Nutrition Examination Surveys (NHANES) [24]. Based on the aforementioned studies and the potential mechanisms underlying the unfavorable health effects of ready-to-eat foods [16, 22], we put forward the hypothesis that a higher intake frequency of ready-to-eat foods was associated with higher odds of depression in US adults.

## Methods

### NHANES – a nationally representative survey

Data for this analysis stems from the NHANES - an ongoing cross-sectional US-based survey designed to capture population-level estimates of the prevalence of health-related outcomes among the noninstitutionalized civilian population [24, 25]. The NHANES uses a complex multistage, stratified, clustered and probability sampling design, and enrolls about 10,000 participants per cycles [24, 25]. The NHANES obtained ethical approval from the National Center for Health Statistics Research (NCHS) Ethics Review Board; all participants provided written informed consent. Four NHANES cycles (2011–2012 through 2017/2018) were combined in this study. The present study adhered to the STROBE (Strengthening the Reporting of Observational Studies in Epidemiology) Guidelines for cross-sectional studies.

### Outcome

Depression was assessed with the PHQ-9 questionnaire [26, 27], a validated screening tool consisting of nine questions with common depression criteria as summarized in the Diagnostic and Statistical Manual of Mental Disorders, Version 5 [26, 28, 29] The PHQ-9 questionnaire has become a measure of reference in depression research and is frequently used in epidemiological studies [28]. Response categories to each PHQ-9 item include “not at all,” “several days,” “more than half the days,” and “nearly every day”, resulting in a score ranging from 0 to 3 points per question [26]. The aggregated maximum score is 27 points whereas the minimum overall score is 0. For this study, we dichotomized this variable and defined PHQ-9-based depression as a total score of 9 points or higher. A comparable yet slightly more conservative cut-off (10 points) may also be found in the literature, and was suggested to maximize combined specificity and sensitivity [30]. More recent data-driven studies, however, recommended using a lower cut-off of 8 points [31, 32]. In light of these findings, we opted for a trade-off between the classical 10-point cut-off and the recently recommended 8-point cut-off, and therefore selected a cut-off of 9 points.

### Exposure

The main exposure of interest in this study was self-reported ready-to-eat food consumption. This information was extracted from the NHANES Diet Behavior & Nutrition section, which included two questions on ready-to-eat foods and ready-to-eat frozen meals/pizza [33]. NHANES participants were asked how frequently they consumed ready-to-eat-foods (such as chicken, sandwiches and cooked vegetables obtained from salad bars and deli counters) in the past 30 days. In addition, participants were queried about the frequency of frozen meals and frozen pizzas they consumed within the past 30 days. Both self-reported frequencies were aggregated in one continuous variable containing the total frequency of ready-to-eat foods within the last 30 days. While primarily entering ready-to-eat food consumption as a continuous variable in all regression models, we also built four intake frequency-based subgroups for stratified analyses and to obtain additional insights into sociodemographic characteristics associated with various degrees of ready-to-eat food consumption. These included: no ready-to-eat food intake, infrequent intake (frequency: between 1 and 14 ready-to-eat foods in 30 days), average intake on at least “every other day” (frequency: between 15 and 29 ready-to-eat foods in 30 days) and at least daily intake on average (frequency: ≥30 ready-to-eat foods in 30 days).

### Covariates

Covariates known to be associated with depression risk were selected based on several other studies in the field of nutritional psychiatry and epidemiology [29, 34–37]. Sociodemographic variables included sex, age, race/ethnicity, educational level and marital status as well as annual household income and poverty level. For the latter, we used the family income to poverty ratio, an NHANES-specific index representing the annual family income adjusted for the family size and the poverty threshold guidelines developed by the US Department of Health and Human Services [38, 39]. The former also considers the living expenses in a particular geographic location. Ratio of family income to poverty ranges from 0 to 5 in the NHANES. For this study, we dichotomized this variable and used a ratio of 1.0 as a cut-off (participants with a ratio below 1.0 were defined as living below the poverty threshold, participants with a ratio above 1.0 were defined as living above poverty threshold). Lifestyle-related covariates included body mass index (BMI, in kg/m²), smoking status (current smoker, former smoker, never smoker), alcohol intake (current drinker, former drinker, never drinker), self-reported diabetes status as well as sedentary behavior (in minutes/day) and daily energy intake (in kcal/day). Nutrient intakes were obtained from the NHANES dietary module and based on a 24-hour dietary recall.

### Inclusion and exclusion criteria

Only NHANES participants with a complete dataset (e.g., no missing values on any variable of interest) were included. Inclusion criteria were as follows: adult participants aged 20 years or older, reliable and plausible energy intake data (≥ 800 kcal/day and ≤ 5000 kcal/day), available anthropometric data and plausible sedentary activity data (at most 17 h of sedentary time, including the total time sitting at work, at home, and when getting to and from places). Participants with special diets (e.g., participants reporting a vegetarian diet or a low-carbohydrate diet) were not considered for this analysis (*n* = 1,584 observations were excluded). Participants who reported a ready-to-eat food intake frequency of more than 100 times within the last 30 days were excluded, as well. This step included the removal of *n* = 18 participants and was performed to highlight full cohort behavior over the casuality of a few outliers. The reported intake frequencies among those 18 individuals were largely deemed unrealistic (e.g., *n* = 14 participants reported consuming ≥ 150 ready-to-eat foods in the last 30 days with the largest frequency reported being *n* = 210).

### Analysis plan and statistical considerations

Statistical calculations were performed using Stata 18 statistical software (StataCorp. 2023. Stata Statistical Software: Release 18. College Station, TX: StataCorp LLC.). Four consecutive NHANES cycles were appended for this analysis (2011/2012 through 2017/2018) and an 8-year-sample weight for interview data was constructed prior to the analysis (wtint8 year = wtint2 year/4). Stata’s “. svyset” and “. svy” commands were used to account for the complex NHANES survey design characteristics and the population weights. Unconditional subclass analyses (preserving the main survey design and providing larger standard errors) were performed when comparing individuals with and without PHQ-9-based depression [34].

Histograms and subpopulation summary statistics were used to assess data distribution. Normally distributed (continuous) variables were described with their mean and 95%-CI in parenthesis. Continuous variables which were not normally distributed were described with their median and Interquartile Range (IQR) in parenthesis. Weighted proportions including CIs were provided for all categorical variables. The NCHS data presentation standards for weighted proportions as outlined by Parker et al. were carefully considered when analyzing the reliability of the estimated proportions [40]. As described earlier [41, 42], we used the user-written post-estimation command “kg_nchs” in Stata and flagged all conspicuous proportions as unreliable [41]. All sample characteristics were shown by PHQ-9-based depression status and by the four pre-defined ready-to-eat food intake frequency categories.

Finally, we constructed multivariate logistic regression models to predict the likelihood of PHQ-9-based depression depending on ready-to-eat food intake frequency. The model building strategies followed the proposed steps by Heeringa et al. in applied survey data analysis [43]. In a first step, we identified factors known to be associated with PHQ-9-based depression in the literature [29, 34, 35]. Associations between candidate predictor variables and PHQ-9-based depression were then assessed using the Rao-Scott F-tests of associations [43]. Only candidate predictors of scientific interest and a bivariate relationship of significance *p* < 0.25 with PHQ-9-based depression were included stepwise in the multivariate logistic model. Close attention was paid to multicollinearity. The importance of the selected predictor variables retained in the model was verified using Wald tests for multiple coefficients. For this, we assessed whether or not the coefficients of *all* predictors changed substantially in the multivariate model (relative to the initial bivariate case). In a first basic model, we adjusted for age (continuous variable), sex (categorical variable, 2 levels) and race/ethnicity (categorical variable, 5 levels). Afterwards, we built model 2 based on the abovementioned technique, in which we additionally adjusted for lifestyle factors. Finally, in model 3, we additionally adjusted for BMI.

In a first step, we entered ready-to-eat food intake frequency as a continuous predictor variable in the respective regression models. Finally, we also built models with ready-to-eat food intake frequency as a categorical predictor variable without modifying the other covariates in the regression model. Results from the regression models were visualized using Stata’s marginsplot function. In a last step, a restricted cubic spline model with 5 knots was used to estimate the overall probability of having PHQ-9-based depression based on ready-to-eat food intake frequency. A *p*-value of less than 0.05 was considered statistically significant.

## Results

The final sample included *n* = 8,689 unweighted observations, thereof *n* = 803 individuals with PHQ-9-based depression. Reasons for in- and exclusion of participants are shown in Supplementary Fig. 1.

Table 1 displays the sample’s characteristics by PHQ-9-based depression status. Participants with and without PHQ-9-based depression differed significantly with regard to sex, race/ethnicity, educational level, marital status, income and BMI as well as with smoking status and diabetes status. NHANES participants with PHQ-9-based depression tended to be female, never married or divorced/separated/widowed and were characterized by a higher proportion of participants with a lower annual household income and by a higher proportion of individuals living below poverty threshold. While median intake frequencies were similar in both groups, the distribution/interquartile ranges of the reported ready-to-eat food frequencies differed significantly between those with PHQ-9-based depression and those without it. The underlying subtle differences are visualized in Supplementary Fig. 2.

**Table 1:** Sample characteristics by PHQ-9-based depression status

	Non-depressed (PHQ9 <9) (*n* =*7*,*886)*	Depressed (PHQ9 ≥9) (*n* =*803)*	*p*-value
**Sex**			***p*** < **0.001**^**a**^
Male	50.39% (48.94-51.83)	38.60% (33.13-44.36) ^*^	
Female	49.61% (48.17-51.06)	61.40% (55.64-66.87) ^*^	
**Age (years)**	46.09 (45.28-46.90)	44.54 (42.73-46.35)	*p* = 0.108 ^b^
**Ethnicity/race**			***p*** **= 0.048** ^**a**^
Mexican American	8.22% (6.29-10.67)	7.88% (5.67-10.86)	
Other Hispanic	5.43% (4.26-6.90)	7.89% (5.55-11.10) ^*^	
Non-Hispanic White	68.45% (64.00-72.59)	64.03% (57.20-70.33)	
Non-Hispanic Black	10.30% (8.29-12.73)	12.64% (9.37-16.84)	
Other Race ^a^	7.60% (6.49-8.87)	7.56% (5.45-10.38)	
**Education level**			***p*** **< 0.001** ^a^
Less than 9th grade	3.74% (3.04-4.58)	7.05% (5.16-9.56) ^*^	
9-11th grade	8.41% (6.98-10.10)	15.55% (12.72-18.88) ^*^	
High school graduate/GED	20.04% (18.35-21.84)	25.71% (22.43-29.29) ^*^	
Some college or AA degree	33.19% (31.35-35.08)	34.69% (30.33-39.33)	
College graduate or above	34.63% (31.51-37.90)	16.99% (12.77-22.26) ^*^	
**Marital status**			***p*** **< 0.001** ^a^
Living with a partner/married	64.93% (62.52-67.27)	47.78% (42.74-52.87) ^*^	
Divorced/separated/widowed/	15.91% (14.71-17.18)	25.10% (21.24-29.41) ^*^	
Never married	19.16% (16.95-21.60)	27.12% (23.21-31.41) ^*^	
**Ratio of family income to poverty**			***p*** **< 0.001** ^a^
< 1 (below poverty threshold)	12.92% (11.30-14.74)	28.71% (25.03-32.68) *	
≥1 (above poverty threshold)	87.08% (85.26-88.70)	71.29% (67.32-74.97) ^*^	
**Annual household income**			***p*** **< 0.001** ^a^
< $20,000	11.85% (10.44-13.41)	26.64% (21.56-32.41) ^*^	
≥ $20,000 & < $75,000	45.69% (42.79-48.62)	54.35% (49.02-59.57) ^*^	
≥ $75,000	42.46% (38.93-46.08)	19.02% (14.74-24.19) ^*^	
**BMI (kg/m²)**	28.42 (28.09-28.75)	30.03 (29.40-30.65)	***p*** **< 0.001** ^**c**^
**BMI category**			***p*** **= 0.001** ^a^
Underweight	1.41% (1.10-1.81)	1.37% (0.61-3.05) ^†^	
Normal weight	31.28% (29.38-33.24)	28.95% (24.60-33.72)	
Overweight	33.82% (32.39-35.29)	26.27% (21.98-31.06) ^*^	
Obesity	33.49% (31.55-35.48)	43.42% (38.64-48.33) ^*^	
**Smoking status**			***p*** **< 0.001** ^a^
Never smoker	59.11% (57.24-60.96)	41.52% (36.56-46.65) ^*^	
Current smoker	17.82% (16.34-19.41)	41.66% (36.82-46.67) ^*^	
Former smoker	23.06% (21.46-24.75)	16.82% (14.05-20.01) ^*^	
**Alcohol intake status**			*p* = 0.571 ^a^
Never drinker	11.69% (9.97-13.66)	10.51% (8.61-13.44)	
Former drinker	10.93% (10.01-11.93)	10.54% (8.23-13.40)	
Current drinker	77.38% (74.96-79.63)	78.95% (75.04-82.93)	
**Minutes sedentary activity/day**	395.86 (386.92-404.81)	411.73 (389.52-433.94)	*p* = 0.181 ^b^
**Diabetes status**			***p*** **< 0.001** ^a^
No	93.70% (92.84-94.47)	88.57% (85.31-91.18) ^*^	
Yes	6.30% (5.53-7.16)	11.43% (8.82-14.69) ^*^	
**Median ready-to-eat food intake (#/30 days)**	2 (5)	2 (6)	***p*** **= 0.010** ^c^
**Energy intake (kcal/day)**	2210.73 (2188.23-2233.24)	2144.51 (2068.80-2220.21)	*p* = 0.091 ^b^

Table 1 shows weighted proportions. The underlying total number of unweighted observations was *n* = 8,689. Continuous variables shown as mean (95%-confidence interval) if normally distributed, or as median (IQR) if not normally distributed. Categorical variables shown as weighted proportions (95%-confidence interval). All weighted proportions can be considered reliable, as per the most recent NCHS Guidelines, except for those marked with an “^†^” symbol, which denotes an unreliable proportion. a = based on Stata’s design-adjusted Rao–Scott test, b = based on regression analyses followed by adjusted Wald tests, c = based on the nonparametric Mann-Whitney U (Wilcoxon rank sum) test; the “*” symbol denotes significant differences in the weighted proportions. AA = Associate of Arts; GED = General Equivalency Degree

Table 2 displays the sample’s characteristics by ready-to-eat food intake frequency category. Compared to those who never ate ready-to-eat foods, those reporting higher intake frequencies were characterized by a lower mean age. Among those eating ready-to-eat foods on average daily or every other day, a higher proportion of individuals living below the poverty threshold was found. Similarly, we found significant associations between income and ready-to-eat food intake frequency. The proportion of individuals characterized by PHQ-9-based depression was highest in those consuming ready-to-eat foods on an average daily basis (16.35%). The distribution of ready-to-eat food intake frequency in the entire sample as well as across the four intake categories is visualized in Supplementary Fig. 3. As displayed in Supplementary Fig. 4, nutrient intakes differed by ready-to-eat food intake frequency category. Compared to participants who did not eat any ready-to-eat-foods, those with a higher ready-to-eat food intake frequency (≥ 1 daily) had a significantly lower dietary intake of fiber (-2.95 g/d (-4.60 – (-1.30)); *p* < 0.001), potassium (-381.64 mg/d (-558.18 – (-205.09)), *p* < 0.001), alpha carotenes (-261.59 mcg/d (-382.98 – (-140.21)), *p* < 0.001), beta-carotenes (-1058.61 mcg/d (-1573.23 – (-543.985)), *p* = 0.001), and vitamin C (-17.26 mg/d (-29.81 – (-4.71)), *p* = 0.046).

**Table 2:** Sample characteristics by ready-to-eat food (RTF) intake frequency category

	RTFs: *n* = 0 (*n=3*,*789)*	RTFs: *n* ≥1 & *n* ≤14 (*n* = *4*,*211)*	RTFs: *n* ≥15 & *n* <30 (*n* = *428)*	RTFs: *n* ≥30 (*n* = *261)*	*p*-value
**Sex**					*p* = 0.704 ^a^
Male	50.28% (48.43-52.12)	48.75% (46.92-50.59)	49.13% (43.44-54.85)	50.66% (42.82-58.46)	
Female	49.72% (47.88-51.57)	51.25% (49.41-53.08)	50.87% (45.15-56.56)	49.34% (41.54-57.18)	
**Age (years)**	47.56 (46.57-48.54)	45.58 (44.70-46.46)	40.25 (37.94-42.56)	43.30 (39.99-46.61)	***p*** **< 0.001** ^b^
**Ethnicity/race**					***p*** **< 0.001** ^a^
Mexican American	11.43% (08.66-14.95)	6.39% (4.91-8.27)	4.69% (2.69-8.07)	5.77% (3.60-9.12) ^*^	
Other Hispanic	7.61% (5.97-9.65)	4.40% (3.36-5.74)	4.11% (2.42-6.91)	5.52% (3.26-9.19) ^*^	
Non-Hispanic White	60.41% (55.28-65.32)	72.65% (68.33-76.58)	75.00% (69.05-80.13)	70.97% (64.24-76.89) ^*^	
Non-Hispanic Black	11.54% (9.20-14.38)	9.89% (7.81-12.45)	7.96% (5.72-10.96)	12.77% (8.93-17.92) ^*^	
Other Race ^a^	9.01% (7.59-10.66)	6.67% (5.53-8.02)	8.25% (6.04-11.16)	4.97% (3.05-8.01) ^*^	
**Education level**					***p*** **< 0.001** ^a^
Less than 9th grade	6.84% (5.53-8.45)	2.17% (1.66-2.84)	1.67% (0.89-3.10) ^†^	5.23% (3.59-7.56) ^*^	
9-11th grade	11.57% (9.56-13.94)	7.48% (6.14-9.07)	5.97% (3.88-9.08)	9.15% (5.60-14.60) ^*^	
High school graduate/GED	19.73% (18.04-21.54)	20.41% (18.01-23.05)	26.23% (20.23-33.27)	21.26% (15.44-28.53)	
Some college or AA degree	29.90% (27.41-32.50)	35.06% (32.83-37.37)	37.85% (31.35-44.83)	36.90% (30.75-43.49) ^*^	
College graduate or above	31.96% (28.32-35.83)	34.87% (31.54-38.36)	28.28% (22.08-35.42)	27.46% (20.56-35.65) ^*^	
**Marital status**					***p*** **< 0.001** ^a^
Living with a partner/married	67.50% (64.98-69.92)	63.60% (60.66-66.44)	44.74% (37.56-52.15)	46.68% (38.02-55.55) ^*^	
Divorced/separated/widowed/	16.94% (15.09-18.97)	16.05% (14.64-17.57)	17.29% (12.26-23.81)	22.86% (16.33-31.04)	
Never married	15.56% (13.28-18.14)	20.35% (17.97-22.95)	37.97% (30.36-46.23)	30.45% (22.63-39.60) ^*^	
**Ratio of family income to poverty**					***p*** **= 0.006** ^a^
< 1 (below poverty threshold)	15.38% (13.38-17.62)	12.69% (10.87-14.78)	17.31% (11.79-24.68)	21.14% (15.22-28.58) ^*^	
≥1 (above poverty threshold)	84.62% (82.38-86.62)	87.31% (85.22-89.13)	82.69% (75.32-88.21)	78.86% (71.42-84.78) ^*^	
**Annual household income**					***p*** **= 0.010** ^a^
< $20,000	14.39% (12.44-16.60)	11.38% (9.71-13.29)	16.76% (12.22-22.56)	19.44% (13.98-26.37) ^*^	
≥ $20,000 & < $75,000	47.27% (43.65-50.91)	46.44% (43.09-49.82)	41.79% (35.02-48.88)	43.54% (34.45-53.07)	
≥ $75,000	38.34% (33.91-42.97)	42.18% (38.71-45.73)	41.45% (33.36-50.03)	37.03% (27.68-47.47)	
**BMI (kg/m²)**	28.55 (28.22-28.87)	28.63 (28.23-29.03)	27.94 (27.11-28.77)	28.48 (27.23-29.74)	*p* = 0.491 ^b^
**BMI category**					***p*** **= 0.006** ^a^
Underweight	1.15% (0.82-1.60)	1.60% (1.18-2.16)	1.49% (0.56-3.88) ^†^	1.17% (0.35-3.81) ^†^	
Normal weight	29.86% (27.55-32.29)	30.84% (28.67-33.09)	40.16% (34.43-46.17)	34.04% (26.82-42.08) ^*^	
Overweight	34.91% (32.90-36.97)	32.73% (31.04-34.46)	23.63% (19.44-28.41)	37.72% (28.49-47.93) ^*^	
Obesity	34.08% (32.06-36.15)	34.84% (32.27-37.50)	34.72% (28.78-41.17)	27.08% (20.64-34.65)	
**Smoking status**					***p*** **< 0.001** ^a^
Never smoker	60.57% (57.79-63.28)	55.93% (53.60-58.24)	59.14% (52.16-65.78)	48.92% (39.82-58.09)	
Current smoker	16.14% (14.30-18.17)	21.18% (19.09-23.43)	26.11% (20.73-32.32)	29.56% (23.26-36.76) ^*^	
Former smoker	23.29% (20.81-25.98)	22.89% (20.78-25.15)	14.75% (10.83-19.76)	21.52% (14.21-31.21) ^*^	
**Alcohol intake status**					***p*** **< 0.001** ^a^
Never drinker	14.40% (12.22-16.90)	10.38% (8.58-12.51)	6.20% (4.34-8.79)	7.68% (5.59-10.46) ^*^	
Former drinker	12.32% (11.13-13.62)	10.03% (8.86-11.33)	9.53% (6.63-13.53)	10.96% (7.15-16.43) ^*^	
Current drinker	73.28% (70.24-76.11)	79.59% (76.96-81.99)	84.26% (79.97-87.78)	81.36% (75.96-85.78) ^*^	
**Minutes sedentary activity/d**	378.78 (367.39-390.17)	406.46 (397.54-415.39)	426.40 (402.50-450.31)	410.03 (378.31-441.74)	***p*** **< 0.001** ^b^
**PHQ-9-based depression**					***p*** **= 0.001** ^a^
No	92.47% (91.37-93.43)	91.96% (90.71-93.06)	88.86% (83.90-92.43)	83.65% (76.49-88.94)	
Yes	7.53% (6.57-8.63)	8.04% (6.94-9.29)	11.14% (7.57-16.10)	16.35% (11.06-23.51)	
**Diabetes status**					***p*** **= 0.005** ^a^
No	92.18% (91.02-93.20)	93.60% (92.34-94.68)	95.49% (92.90-97.17)	97.13% (94.90-98.40) ^*^	
Yes	7.82% (6.80-8.90)	6.40% (5.32-7.66)	4.51% (2.83-7.10)	2.87% (1.60-5.10) ^*,†^	
**Energy intake (kcal/day)**	2165.31 (2136.61-2194.01)	2220.35 (2186.38-2254.32)	2328.54 (2244.21-2412.87)	2211.92 (2069.66-2354.18)	***p*** **= 0.002** ^b^
**Median ready-to-eat food intake frequency (#/30 d)**	0 (0)	3 (4)	19 (5)	34 (30)	***p*** **< 0.001** ^c^

Table 2 shows weighted proportions. The underlying total number of unweighted observations was *n* = 8,689. Continuous variables shown as mean (95%-confidence interval) if normally distributed, or as median (IQR) if not normally distributed. Categorical variables shown as weighted proportions (95%-confidence interval). All weighted proportions can be considered reliable, as per recent NCHS Guidelines, except for those individual proportions marked with an “^†^” symbol, which denotes an unreliable proportion. a = based on Stata’s design-adjusted Rao–Scott test, b = based on regression analyses followed by adjusted Wald tests, c = based on the nonparametric Kruskal-Wallis equality-of-populations rank test; the “*” symbol denotes significant differences in the weighted proportions. AA = Associate of Arts; GED = General Equivalency Degree; RTF = Ready-to-Eat Food

Table 3 displays multivariate logistic regression models ascertaining the effects of ready-to-eat food intake frequency on PHQ-9-based depression. At first, we entered ready-to-eat food intake frequency as a continuous variable (Table 3, top row). For each additional ready-to-eat food reported, the odds of having PHQ-9 based depression increased significantly by a factor of 1.017, when adjusting for sex, age, and race/ethnicity (CI: 1.008–1.027; *p* = 0.001). When additionally adjusting for other lifestyle-related covariates including educational level smoking and alcohol status, poverty level and sedentary activity time, these findings remained essentially unchanged (OR: 1.014; CI: 1.002–1.026; *p* = 0.022). Similarly, adjusting for BMI in model 3 did not alter these findings. In a second step, we entered ready-to-eat food intake frequency as a categorical variable, using the four categories explained earlier. Compared to those who did not report any ready-to-eat foods within the last 30 days, those with a frequency of *n* = 30 or more had a significantly higher likelihood of depression (OR: 2.40, CI: 1.51–3.83; *p* < 0.001) when adjusting for sex, age, and race/ethnicity in model 1 (Table 3, bottom). When adjusting for additional covariates in model 3, the likelihood for PHQ-9-based depression was slightly lower but remained significant (OR: 2.02, CI: 1.18–3.43; *p* = 0.011). The contrast between the highest and the lowest ready-to-eat food intake frequency category was significant at a *p*-value of 0.011: 0.70 (CI: 0.17–1.23).

**Table 3:** Logistic regression models for the association between the reported ready-to-eat food (RTF) intake frequency and PHQ-9-based depression

Multivariate regression model with ready-to-eat food intake frequency in the past 30 days as a continuous independent variable (# of RTF)									
	Model I			Model II			Model III		
	OR	95%-CI	p-value	OR	95%-CI	p-value	OR	95%-CI	p-value
# of RTFs	1.017	1.008-1.027	**0.001**	1.014	1.002-1.026	**0.022**	1.014	1.002-1.026	**0.024**
Multivariate regression model with ready-to-eat food intake frequency in the past 30 days as a categorical independent variable									
	OR	95%-CI	p-value	OR	95%-CI	p-value	OR	95%-CI	p-value
RTFs: *n* = 0	1.00 (Ref.)			1.00 (Ref.)			1.00 (Ref.)		
RTFs: *n* ≥1 & *n* ≤14	1.08	0.87-1.35	0.490	1.06	0.84-1.32	0.635	1.05	0.84-1.31	0.665
RTFs: *n* ≥15 & *n* <30	1.53	0.96-2.44	0.074	1.35	0.82-2.21	0.233	1.35	0.82-2.22	0.229
RTFs: *n* ≥30	2.40	1.51-3.83	**<0.000**	2.01	1.18-3.42	**0.011**	2.02	1.18-3.43	**0.011**

Table 3 displays multivariate regression model with ready-to-eat food intake frequency in the past 30 days entered as a continuous independent variable (top) and as a categorical variable (bottom). Model I adjusted for age (continuous), sex (categorical, 2 levels) and race/ethnicity (categorical, 5 levels). Model 2 additionally adjusts for education level (categorical, 5 levels), smoking status (categorical, 3 levels), alcohol status (categorical, 3 levels), poverty level (categorical, 2 levels) and minutes of sedentary activity per day (continuous). Model 3 includes all independent variables from model 2 and additionally adjusts for the body mass index (continuous variables)

**Fig. 1:**
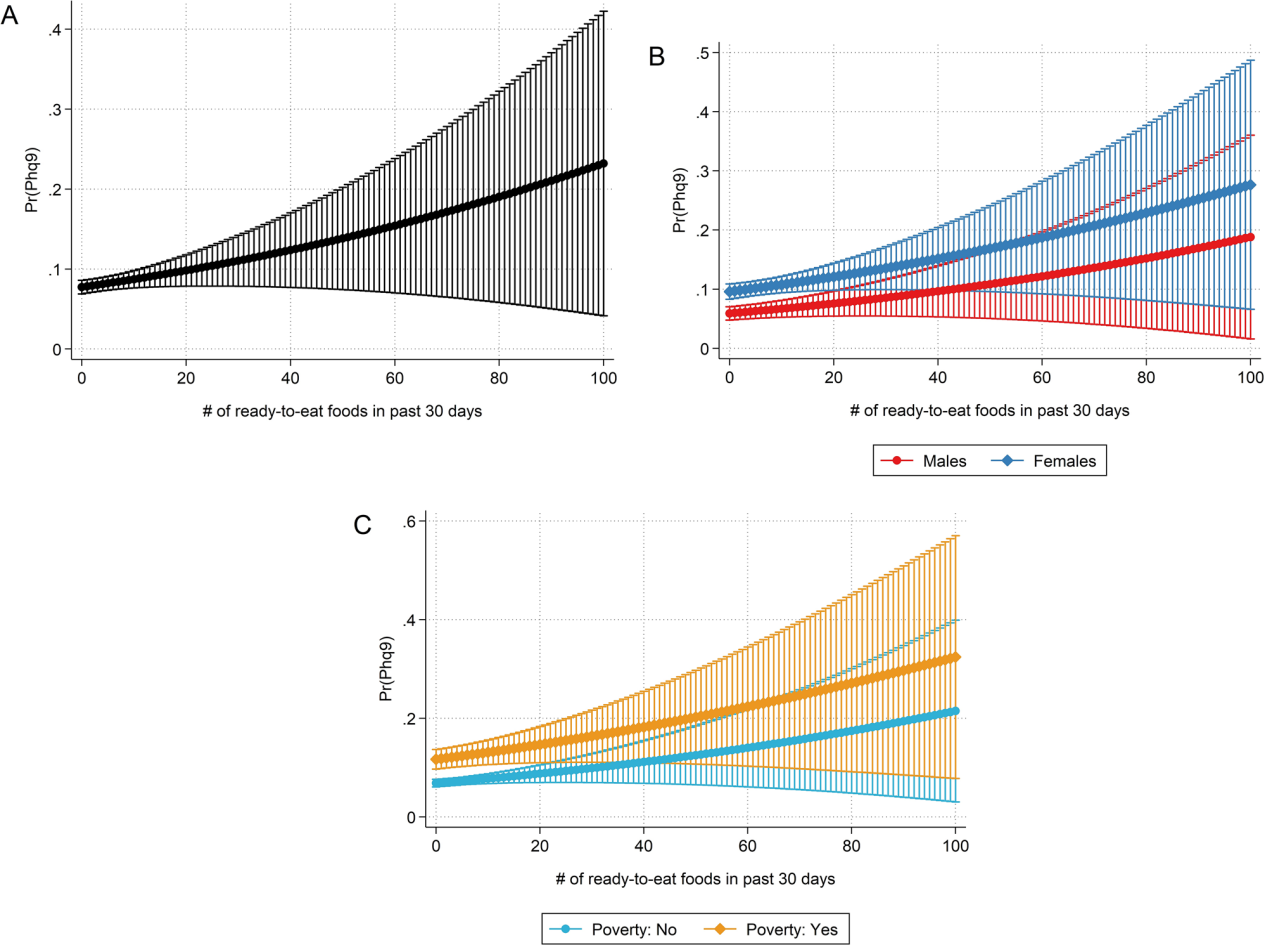
The association between an increased ready-to-eat food consumption and the predicted probability of depression. The probability of having PHQ-9 based depression increases with higher ready-to-eat food intake frequency. The displayed marginsplot in panel **A** is based on a multivariate regression model (model III), which adjusts for sociodemographic (age, sex, race/ethnicity, education, income) and lifestyle factors (smoking status, alcohol intake, sedentary activity, and body mass index). Panel **B** displays the predicted probabilities of depression over an intake frequency of 0 to 100 ready-eat-foods in 30 days for males and females based on the same model. Likewise, Panel **C** shows the predicted probabilities of depression depending on poverty status (yes vs. no) based on the same model

Results from regression model 3 were visualized in Fig. 1, which shows the predicted probabilities of depression over an intake frequency of 0 to 100 ready-eat-foods in 30 days (panel A). Based on the same model, panel B shows the predicted probabilities of depression for males and females. Similarly, panel C shows the predicted probabilities of depression depending on poverty status.

**Fig. 2:**
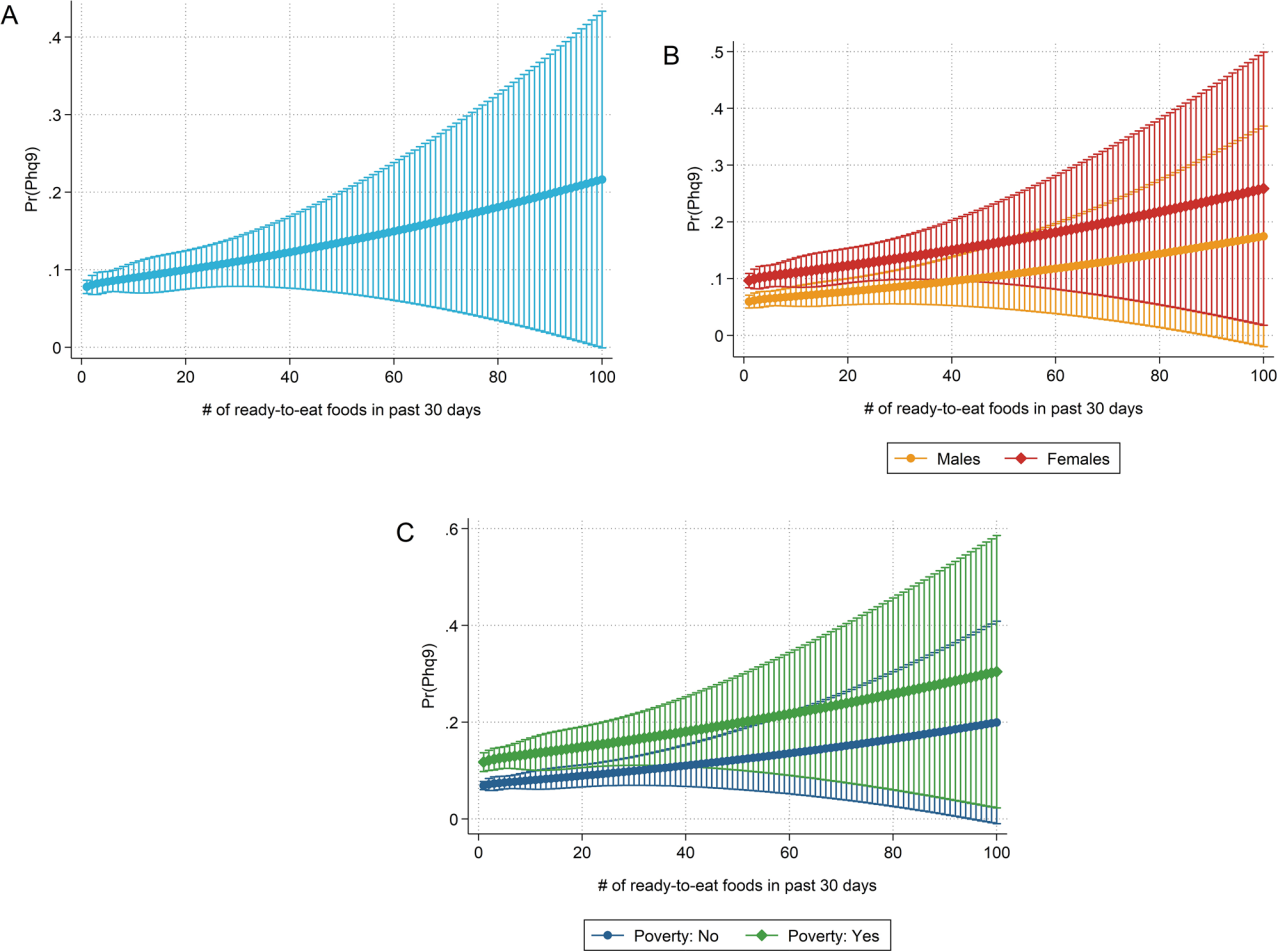
The association between an increased ready-to-eat food consumption and the predicted probability of depression based on a restricted cubic spline regression model with 5 knots. The probability of having PHQ-9 based depression increases with a higher intake frequency of ready-to-eat foods consumption. The displayed marginsplot in panel **A** is based on a restricted cubic spline regression model with 5 knots, adjusting for sociodemographic (age, sex, race/ethnicity, education, income) and lifestyle factors (smoking status, alcohol intake, sedentary activity, and body mass index). Panels **B** and **C** show the predicted probabilities of depression for males and females (panel **A**) and based on poverty status (panel **B**), respectively

Figure 1 suggested a potential non-linear relationship between ready-to-eat food intake frequency and the probability of PHQ-9 based depression. In light of these findings and based on the data distribution, we also employed a restricted cubic spline model with 5 knots to estimate the overall probability of having PHQ-9-based depression based on the ready-to-eat food intake frequency. The results are visualized in Fig. 2. Except for the cubic splines, no changes were made to model 3 with regard to the other covariates. Similarly to Fig. 1, predicted probabilities are also displayed for males and females and depending on poverty status.

## Discussion

Ready-to-eat food intake frequency increased the odds for PHQ-9-based depression in adults aged 20 years or older in this US-based cross-sectional study. For each additional ready-to-eat food, the odds increased by a factor of 1.014 (CI: 1.002–1.026; *p* = 0.022) after adjustment for sociodemographic and lifestyle characteristics. Given the high prevalence and popularity of ready-to-eat foods in the US [23], these findings have important implications from a public health nutrition perspective.

Ready-to-eat foods maximize convenience and require minimal to no preparation (except heating), often at the expense of nutritional quality. Such foods were frequently characterized by a high (saturated and trans) fat and sodium content while being low in vitamins and minerals due to their high processing degree [18, 19]. These nutritional properties have been linked to neuroinflammation and depression risk [16]. Marx et al. summarized potential pathways linking a high intake of high-fat processed foods with depression and emphasized a complex interplay involving neuroinflammation, oxidative stress, unfavorable gut microbiota alterations as well as mitochondrial dysfunction due to a high intake of processed foods [16]. In the context of ultraprocessed foods, Samuthpongtorn and others suggested that artificial sweeteners could be involved in the etiopathogenesis of depression by eliciting purinergic transmission in the brain [36, 44, 45]. Further to that, obesity subsequent to an excessive intake of high-energy foods has also been highlighted as a potential cause of mood disorders [16].

In this context, it is important to emphasize that an obesity-centered mediation analysis on depression and fast food intake revealed that obesity plays an important role while not fully mediating the relationship between both entities [29]. The herein presented findings are somewhat similar, given that ready-to-eat food intake frequency significantly increased the likelihood for depression even after adjusting for BMI. While a detailed discussion of potentially involved pathways is beyond the scope of this article, it is of utmost importance to discuss the potential public health implications of our findings.

Previous studies emphasized that public health nutrition strategies should be targeted at modifying the exposure to ready-to-eat food environments [46]. It is noteworthy that public health policies and practice that simply involve providing information and that solely aim at transferring nutritional knowledge are rather unlikely to be effective [47]. To the contrary, effective interventions that promote healthier ready-to‐eat meals sold by food outlets should either restrict choice or guide customers through incentives/disincentives [47]. While ethically debatable, it has been suggested that ‘intrusive’ interventions that restricted or guided choice showed a more favorable impact on food‐outlet‐level and customer‐level outcomes when compared to pure informational campaigns [47]. Beyond that, whole‐system changes across the out‐of‐home food sector would require concerted efforts and joined up action across a range of public and private sector organizations that must be supported by political will and, ideally, public opinion [48–50].

Independent of the public health implications, it is noteworthy that a consistent decrease in fast-food consumption has been shown to be effective when it comes to weight loss and mental health improvements [51, 52]. While likely applying to ready-to-eat foods, as well, further research in this area is warranted.

The present study has multiple strengths and weaknesses worth further consideration. Strengths include the large sample size, the underlying NHANES dataset with its complex multistage, stratified, clustered and probability sampling design that allows for nationally-representative population level estimations, and, finally, the consideration of numerous covariates known to be associated with depression risk. The coherent findings when entering ready-to-eat food intake frequency as a continuous and as a categorical predictor into the regression models further strengthens confidence in the obtained results. As for the weaknesses, it must be emphasized that the cross-sectional study design does not allow for any causal inferences. Ready-to-eat food intake frequency was self-reported and this information is thus subject to report and recall bias. Intake frequencies do not necessarily correlate with the amount of ready-to-eat foods or number of days when ready-to-eat foods were consumed. It is possible that more than one ready-to-eat food was consumed on a reported occasion. Likewise, the temporal distribution is just an approximation, and consuming more than 30 ready-to-eat foods within 30 days does not necessarily imply daily consumption.

Although the PHQ-9 questionnaire has become a measure of reference in depression research [28], depression remains a clinical diagnosis and PHQ-9 results alone may not be sufficient to establish this diagnosis [53]. Results must thus be interpreted carefully. Similarly, one cannot rule out reversed causality when interpreting the herein presented results. Finally, we acknowledge that the query for the frequency of ready-to-eat foods consumed over the past 30 days may not reflect long-term eating habits. Likewise, the PHQ-9 questionnaire does not cover long-term depression risk. Despite these potential limitations, we believe that our results add to the literature and are largely in line with the findings of other studies on ready-to-eat, ready-to-heat and ultraprocessed food intake and the risk of depression [54, 55].

The herein presented associations should nevertheless be explored in future studies in different contexts and other populations. Future studies could also attempt to dissect the ready-to-eat food category further, potentially examining the association between individual food types (e.g., ready-made sandwiches vs. frozen ready-made meals) and depression. This may allow for additional insights, as the ready-to-eat food category is large and encompasses numerous different food items with varying properties. Due to the NHANES Diet Behavior & Nutrition section data structure, we were not able to differentiate between less processed foods, such as pre-prepared fruits, and highly processed packaged meals.

## Conclusions

Ready-to-eat food intake increased the odds for PHQ-9-based depression in adults aged 20 years or older in this US-based cross-sectional study. For each additional ready-to-eat food, the odds for depression increased by a factor of 1.014 (CI: 1.002–1.026; *p* = 0.022), even after adjustment for sociodemographic covariates and BMI. Given the high prevalence and popularity of ready-to-eat foods in the US, these findings have important implications from a public health nutrition perspective, and warrant targeted interventions to promote healthier meals sold by food outlets. From a scientific perspective, it is essential to examine the association between individual ready-to-eat food types (e.g., ready-made sandwiches vs. frozen ready-made meals) and depression in greater detail.

## Electronic supplementary material

Below is the link to the electronic supplementary material.

Supplementary Material 1: Supplementary Figure 1: Stepwise participant inclusion flowchart with detailed reasons for in- and exclusion of participants: Legend: The final sample comprised n = 8,689 unweighted observations.

Supplementary Material 2: Supplementary Figure 2: Histograms showing ready-to-eat foods intake frequency by PHQ-9-based depression status. Legend: Histograms depict the frequency of ready-to-eat foods reported within the last 30 days. Panel A depicts individuals without depression (n = 7,886), whereas panel B shows the frequency of reported ready-to-eat food intakes in individuals with PHQ-9-based depression (n = 803).

Supplementary Material 3: Supplementary Figure 3: Histograms showing ready-to-eat foods intake frequency in the entire sample and in the 4 pre-defined intake groups. Legend: Histograms depict the frequency of ready-to-eat foods reported within the last 30 days. Panel A depicts the entire sample with n = 8,689 unweighted observations. Panel B shows intake frequencies in individuals reporting ready-to-eat food intake frequencies between 1 and 14 within the last 30 days (n = 4,211). Panel C displays those reporting an average ready-to-eat food intake frequency of at least every other day but not every day (n = 428). Panel D displays intake frequencies in individuals reporting a ready-to-eat food intake frequency ≥30 (n = 261).

Supplementary Material 4: Supplementary Figure 4: Marginal predicted nutrient intake values by ready-to-eat food intake frequency category. Legend: All panels are based on n = 8,689 unweighted observations.

## Data Availability

Data is publicly available online (https://wwwn.cdc.gov/nchs/nhanes/Default.aspx). The datasets used and analyzed during the current study are available from the corresponding author on reasonable request.

## Declarations

### Prior presentation

This work has not been published before; it is not under consideration for publication anywhere else. This work been approved by all co-authors.

## Abbreviations

BMI: Body Mass Index
CI: Confidence Interval
NCHS: National Center for Health Statistics
NHANES: National Health and Nutrition Examination Surveys
OR: Odds Ratio
PHQ: Patient Health Questionnaire
