# Supplementary figures and images for: Ready-to-eat food intake associates with PHQ-9-based depression in US adults: a cross-sectional study
Source: BMC Public Health. 2025 May 13;25:1755. doi: 10.1186/s12889-025-22930-x (PMC12070559; doi:10.1186/s12889-025-22930-x)

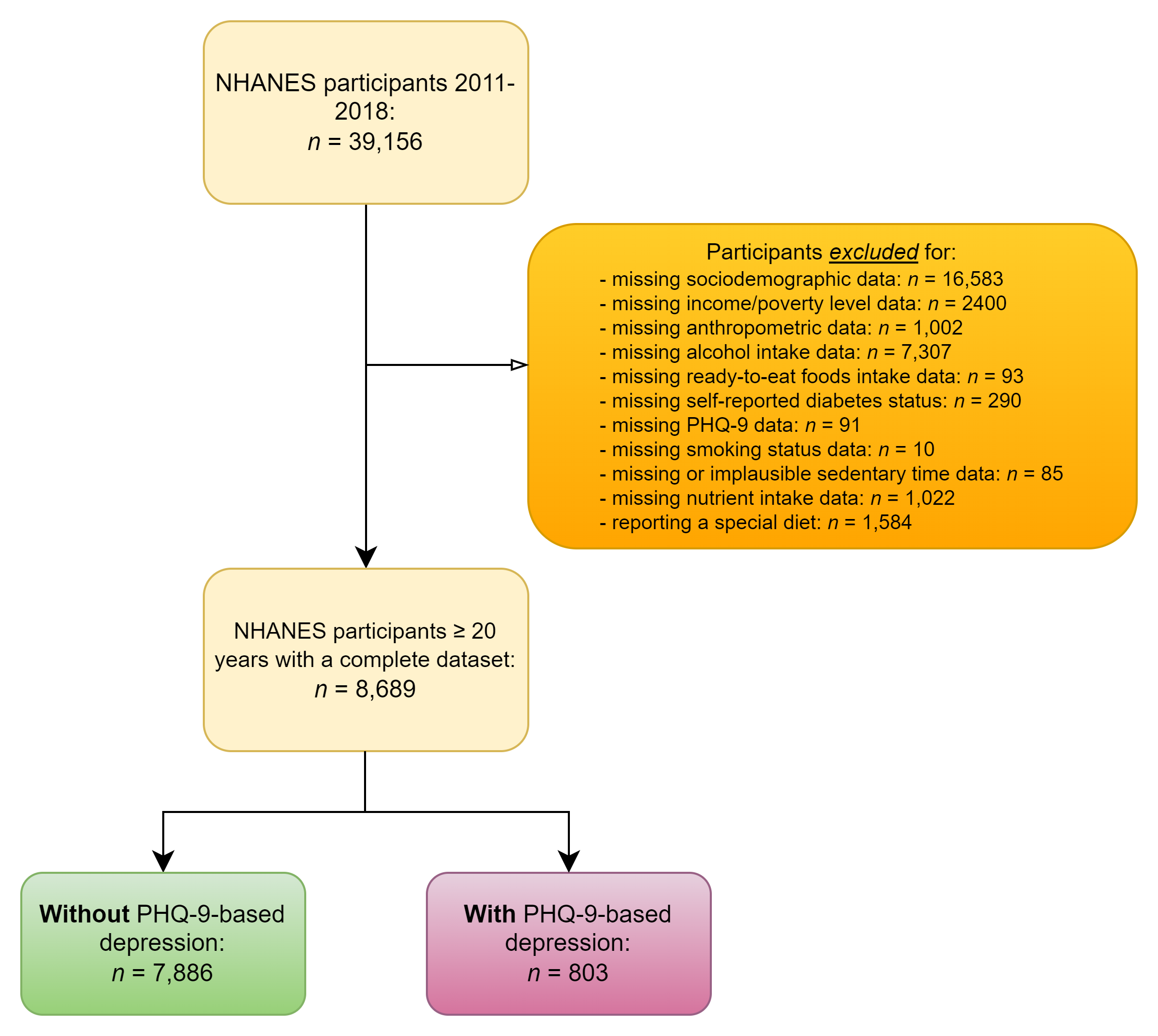

Supplement: Supplementary file 1 — Supplementary Material 1: Supplementary Figure 1: Stepwise participant inclusion flowchart with detailed reasons for in- and exclusion of participants: Legend: The final sample comprised n = 8,689 unweighted observations. [file 12889_2025_22930_MOESM1_ESM.png]

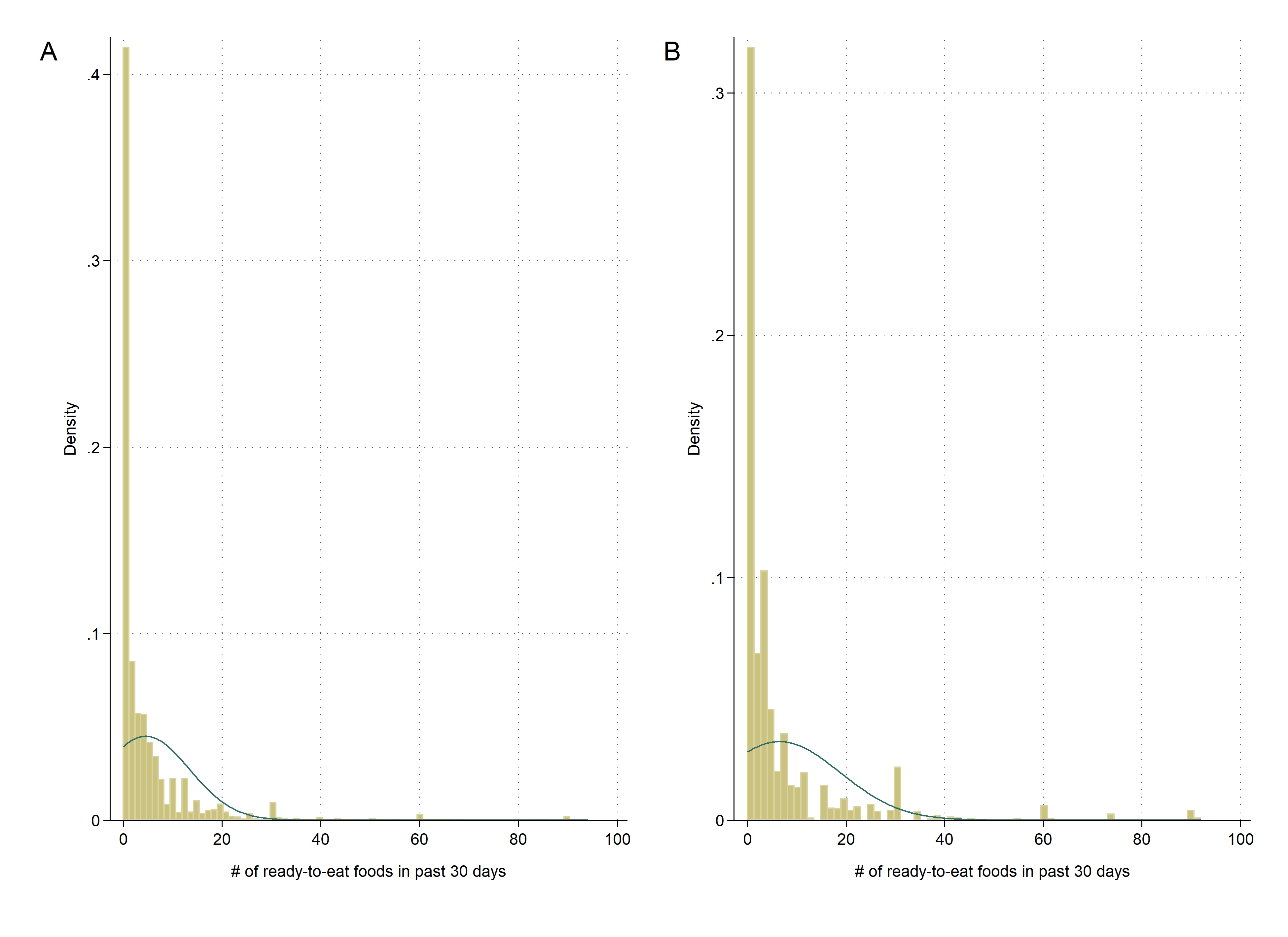

Supplement: Supplementary file 2 — Supplementary Material 2: Supplementary Figure 2: Histograms showing ready-to-eat foods intake frequency by PHQ-9-based depression status. Legend: Histograms depict the frequency of ready-to-eat foods reported within the last 30 days. Panel A depicts individuals without depression (n = 7,886), whereas panel B shows the frequency of reported ready-to-eat food intakes in individuals with PHQ-9-based depression (n = 803). [file 12889_2025_22930_MOESM2_ESM.jpg]

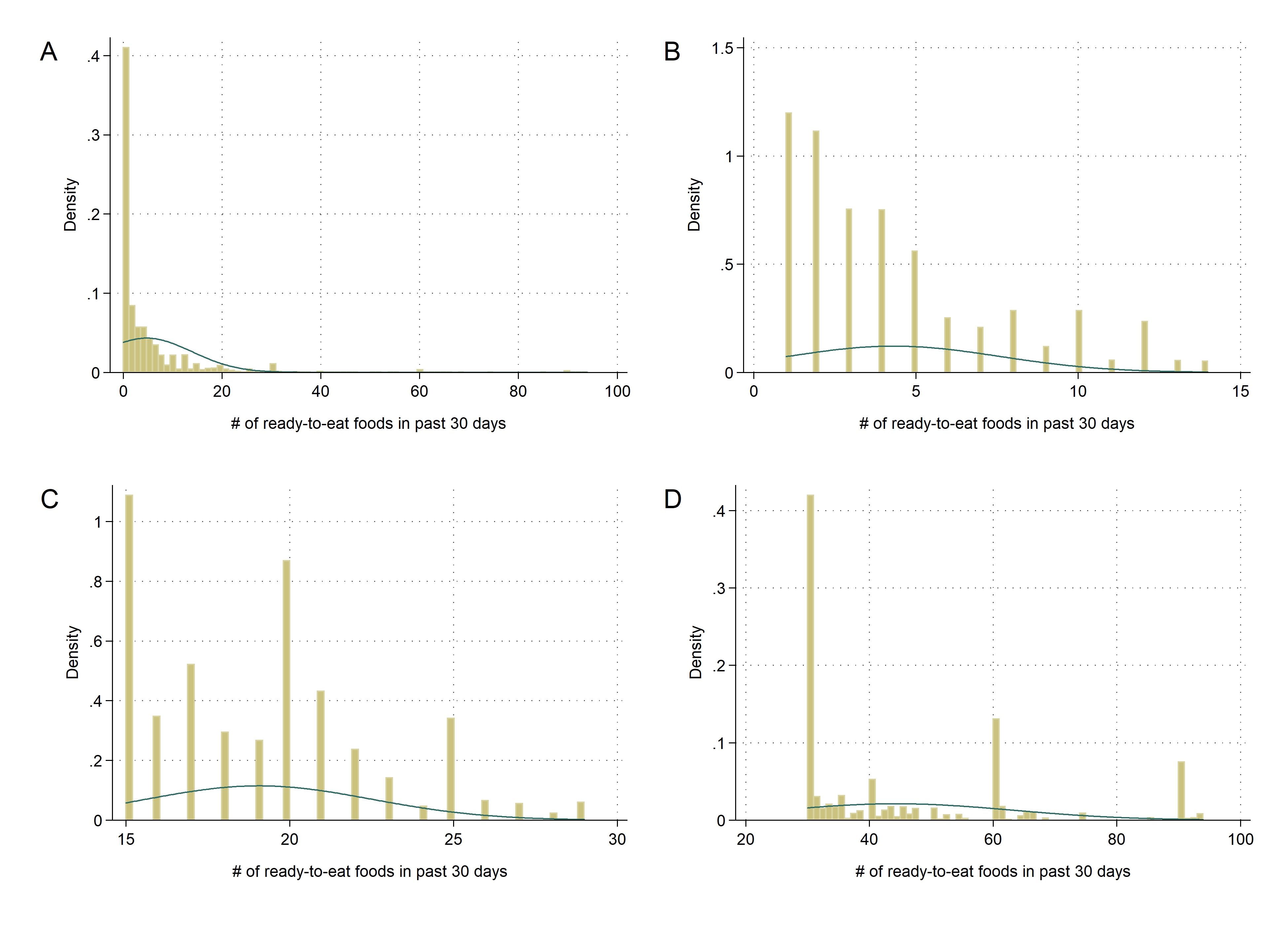

Supplement: Supplementary file 3 — Supplementary Material 3: Supplementary Figure 3: Histograms showing ready-to-eat foods intake frequency in the entire sample and in the 4 pre-defined intake groups. Legend: Histograms depict the frequency of ready-to-eat foods reported within the last 30 days. Panel A depicts the entire sample with n = 8,689 unweighted observations. Panel B shows intake frequencies in individuals reporting ready-to-eat food intake frequencies between 1 and 14 within the last 30 days (n = 4,211). Panel C displays those reporting an average ready-to-eat food intake frequency of at least every other day but not every day (n = 428). Panel D displays intake frequencies in individuals reporting a ready-to-eat food intake frequency ≥30 (n = 261). [file 12889_2025_22930_MOESM3_ESM.jpg]

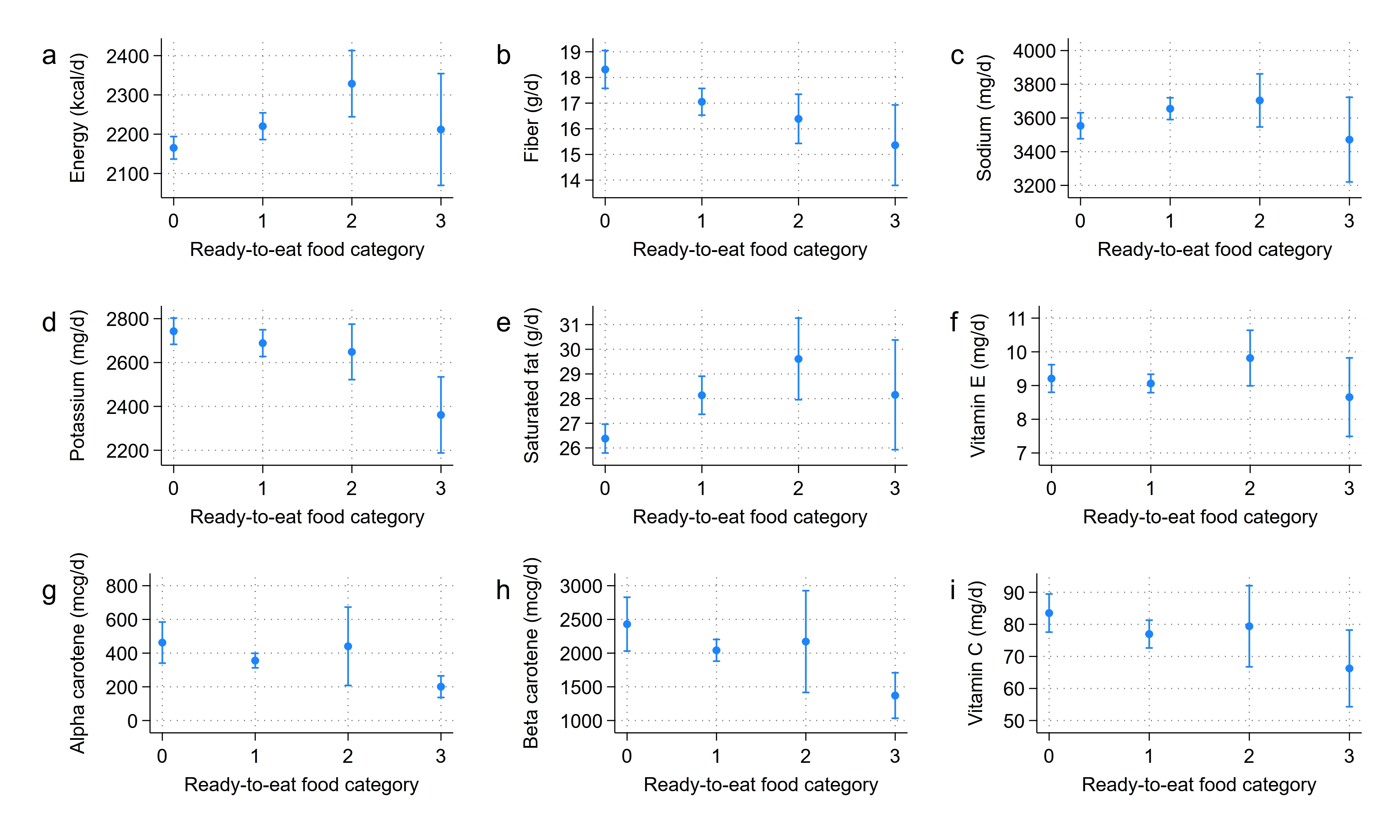

Supplement: Supplementary file 4 — Supplementary Material 4: Supplementary Figure 4: Marginal predicted nutrient intake values by ready-to-eat food intake frequency category. Legend: All panels are based on n = 8,689 unweighted observations. [file 12889_2025_22930_MOESM4_ESM.jpg]
